# Development and Validation of the Camouflaging Autistic Traits Questionnaire (CAT-Q)

**DOI:** 10.1007/s10803-018-3792-6

**Published:** 2018-10-25

**Authors:** Laura Hull, William Mandy, Meng-Chuan Lai, Simon Baron-Cohen, Carrie Allison, Paula Smith, K. V. Petrides

**Affiliations:** 1 Research Department of Clinical, Educational & Health Psychology, University College London, London, UK; 2 Child and Youth Mental Health Collaborative, Centre for Addiction and Mental Health and The Hospital for Sick Children, Department of Psychiatry, University of Toronto, Toronto, ON Canada; 3 Autism Research Centre, Department of Psychiatry, University of Cambridge, Cambridge, UK; 4 Department of Psychiatry, National Taiwan University Hospital and College of Medicine, Taipei, Taiwan; 5 London Psychometric Laboratory, University College London, London, UK; 6 Department of Psychology, University College London, 26 Bedford Way, London, WC1H 0AP UK

**Keywords:** Autism, Camouflaging, Masking, Compensation, Coping Strategies

## Abstract

There currently exist no self-report measures of social camouflaging behaviours (strategies used to compensate for or mask autistic characteristics during social interactions). The Camouflaging Autistic Traits Questionnaire (CAT-Q) was developed from autistic adults’ experiences of camouflaging, and was administered online to 354 autistic and 478 non-autistic adults. Exploratory factor analysis suggested three factors, comprising of 25 items in total. Good model fit was demonstrated through confirmatory factor analysis, with measurement invariance analyses demonstrating equivalent factor structures across gender and diagnostic group. Internal consistency (α = 0.94) and preliminary test–retest reliability (r = 0.77) were acceptable. Convergent validity was demonstrated through comparison with measures of autistic traits, wellbeing, anxiety, and depression. The present study provides robust psychometric support for the CAT-Q.

**Electronic supplementary material:**

The online version of this article (10.1007/s10803-018-3792-6) contains supplementary material, which is available to authorized users.

Social camouflaging is defined as the use of strategies by autistic people to minimise the visibility of their autism during social situations (Lai et al. 2011). This topic has recently come to the attention of researchers, as recognised by the call for clinicians to be aware of masking or coping behaviours when assessing autism in the newly released 11th edition of the International Classification of Diseases (Zeldovich 2017), but has been described by autistic people and clinicians for many years. It may be a widespread and important phenomenon in autism, especially in intellectually able individuals. We note here that, following preferences from a majority of the autism community (Kenny et al. 2015), we use identity-first language in this paper (e.g. ‘autistic person’) while recognising that some individuals prefer the use of person-first language (e.g. ‘person with autism’). Social camouflaging encompasses an explicit effort to ‘mask’ or ‘compensate’ for autistic characteristics; and to use conscious or unconscious techniques which result in a less autistic behavioural presentation (Hull et al. 2017; Lai et al. 2017; Livingston and Happé 2017). Examples of camouflaging behaviours described in the current literature include forcing oneself to make eye contact during a social interaction, or pretending that one is doing so by looking at the space between someone’s eyes or at the tip of their nose, or using working memory strategies to develop a list of appropriate topics for conversation. Camouflaging is driven by the desire to ‘fit in’ so as to appear non-autistic, and to form relationships with others, which may be harder to achieve when the person presents autistic behaviour (Hull et al. 2017).

The concept of social camouflaging was first investigated through qualitative research with autistic girls and women, looking in particular at reasons why these individuals may not be diagnosed until later in life. Themes identified in this research include the concept of ‘masking’, or portraying a non-autistic persona (Bargiela et al. 2016; Tierney et al. 2016), and the idea that through copying others and controlling behavioural expression, autistic girls and women could compensate for some of the social and communication difficulties they experienced (Dean et al. 2017). This qualitative research suggested that there may be some negative consequences of camouflaging. These include links to heightened stress or mental health conditions such as depression, and reduced access to clinical support and services as a result of difficulties being hidden (Cage et al. 2017; Head et al. 2014). Autistic females’ camouflaging may even account for the later and less frequent diagnoses of females than males with the same autistic characteristics (Begeer et al. 2013; Giarelli et al. 2010; Rutherford et al. 2016). In addition, some qualitative research that has begun to look at the experiences of camouflaging amongst autistic men suggests that while both men and women may camouflage their autism, there might be gendered differences in both the techniques used and the consequences of camouflaging (Hull et al. 2017).

This qualitative research has offered new insights into under-investigated social behaviours in autism, and has raised important questions to address: Who, among the many different autistic people, camouflages their autism? Do autistic girls and women camouflage more than boys and men, and does this partly account for gender disparities in the rate and timing of diagnosis (Begeer et al. 2013; Loomes et al. 2017)? What is the relationship between camouflaging and mental health outcomes? Quantitative investigation of these questions has to date been hindered by the challenges of accurately measuring camouflaging.

## Measures of Social Camouflaging

In recent years there have been some attempts to quantify social camouflaging by autistic people. The resultant instruments reflect different ways of defining and operationalising camouflaging, leading to some overlap but also some discrepancies in how camouflaging behaviours are measured.

Livingston and Happé (2017) suggest that camouflaging is a component of compensation, the “processes contributing to improved behavioural presentation of a neurodevelopmental disorder, despite persisting core deficit(s) at cognitive and/or neurobiological levels” (p. 8), and hence should be measured at the behavioural, cognitive, and neurobiological levels. We call these approaches to measuring camouflaging ‘discrepancy methods’, as they seek to measure the gap between how autistic a person is (‘internal autistic status’) and their overt behaviours (‘external autistic presentation’). This approach was used by Lai et al. (2017), who measured the discrepancy between self-reported autistic traits on the Autism Spectrum Quotient (AQ) as well as social cognitive abilities on the Reading the Mind in the Eyes test (RMET) (internal autistic status) and scores on the Autism Diagnostic Observation Schedule (ADOS) (external autistic presentation). The study found a greater AQ/RMET-ADOS discrepancy score for female than male participants, and that higher discrepancy scores were associated with greater depressive symptoms for men, but not for women.

A similar result was found by Ratto et al. (2017), where autistic females with higher IQ were less likely to meet diagnostic criteria on the Autism Diagnostic Interview (Revised) than males, despite being matched on ADOS scores and having higher levels of parent-report functioning difficulties than males. Other studies have demonstrated autistic females’ greater use of camouflaging strategies during communication than males, whether through gesture (Rynkiewicz et al. 2016), or filling pauses in conversation (Parish-Morris et al. 2017), despite overall comparable social skills. These methods measure camouflaging by identifying discrepancies between different measures of social ability or autistic characteristics, such that individuals (especially females) appear less autistic in some settings yet still meet autism diagnostic criteria in others.

A strength of these discrepancy approaches is their conceptual rigour, as they seek to operationalize the key feature of camouflaging; that it is a dissociation between an individual’s experience of being autistic and the behaviours they portray to the outside world. In addition, using autism assessment tools as a measure of external autism presentation demonstrates the impact camouflaging can have in a clinical setting, especially for autistic women. However, a key disadvantage is that this approach relies upon an index of how autistic a person is (i.e., their internal autistic status) independent of their behavioural presentation. Given that autism is currently conceptualised at the behavioural level because there are no reliable biomarkers for the condition (American Psychiatric Association 2013; Loth et al. 2015), this represents a significant conceptual and practical challenge. Performance on tests of cognition relevant to autism, or scores on self-reported measures of autism traits can only ever be a proxy measure of internal autistic status: we currently have no way to identify how autistic an individual is meaningfully and accurately.

In addition, the measurement of camouflaging using discrepancy approaches does not allow for unsuccessful camouflaging attempts to be assessed. Some autistic people may use a variety of strategies in an attempt to appear less autistic to others, but these may be only partially or not at all successful. This is especially important when considering the evidence for a link between self-reported camouflaging and poor mental health (Cage et al. 2017; Hull et al. 2017). If individuals attempt to camouflage but are ultimately unsuccessful this may further increase the social, emotional, and psychological harm resulting from their camouflaging efforts.

An alternative to the discrepancy approaches described above is one based on observational recognition of camouflaging; measuring the specific behaviours and experiences which represent camouflaging. Such ‘observational/reflective methods’ circumvent the limitation of being unable to measure an individual’s internal autistic state. Camouflaging can be measured consistently and compared between individuals, and behaviours can be identified regardless of how successful they are. In other words, identification of camouflaging is not reliant on either a proxy measure of internal autistic status, or the need to display a typical social presentation.

Dean et al. (2017) used an observational/reflective approach to identify camouflaging strategies used by autistic girls when interacting with peers through behavioural observation. Behavioural techniques, such as standing near to peers who are interacting, but not actually engaging in with them, were classified as camouflaging strategies, and were observed more in autistic girls than in autistic boys or non-autistic girls, in a school setting. This led to the superficial appearance of successful social interaction, but did not actually result in friendships or sustained engagement for the autistic girls using these techniques. Dean et al. operationalisation of camouflaging is based on the idea of blending into the social environment, a strength of which is that the need to camouflage may vary depending on the situation. In addition, this definition of camouflaging emphasises that camouflaging behaviours may be learned or mimicked from non-autistic peers.

This approach to camouflaging has the advantage of allowing for variation in camouflaging behaviours and their success. Techniques learned and used in some situations may not be successful in others, and an individual’s overall camouflaging ability may partly depend on their ability to adapt to different situations. The cognitive flexibility enabling this has already been suggested as one explanation for autistic girls’ superficially higher social skills (Lehnhardt et al. 2015). However, this measure of camouflaging is based on non-autistic observers’ ideas of what camouflaging looks like. Intentions and behaviours of camouflaging which clinicians and researchers may not be aware of, but which may form an important part of autistic individuals’ camouflaging strategies, have not yet been measured.

## Self-Reported Measurement of Camouflaging

Another observational/reflective approach to the operationalisation of camouflaging addresses some of these remaining issues: asking autistic people themselves about their camouflaging behaviours. Here, camouflaging is conceptualised based on the reported experiences of individuals who have (and have not) camouflaged their autism, and the behaviours and intentions described by these individuals are used to develop a list of camouflaging strategies to measure. Autistic individuals can then report directly on their own camouflaging behaviours, identifying strategies and intentions that might not be visible to an observer without in-depth discussion with the autistic person themselves.

This self-report method, based on an observational/reflective approach, has several strengths. First, identifying camouflaging behaviours based on strategies reported by autistic individuals reduces the potential for introducing bias via researchers’ and clinicians’ perceptions of autistic behaviours and abilities. Autistic adults have previously reported being told by clinicians that their ability to camouflage (for example, by making or appearing to make eye contact) meant they could not be autistic, despite meeting autism diagnostic criteria in other ways (Hull et al. 2017). Clinicians and researchers may only observe autistic individuals in one structured and limited situation and so may not identify certain behaviours as camouflaging strategies, whereas autistic individuals and those who know them well have a unique insight into their own behaviours across a variety of situations. Second, self-report measures of camouflaging allow for operationalisation of the attempt to camouflage—the intention put into camouflaging autistic characteristics, and the techniques used, which may not result in any observable external change for someone who does not know the person well.

Both the discrepancy and observational/reflective approaches described above offer ways to define and therefore measure camouflaging in autism. All the methods used or suggested have their own strengths and weaknesses, thus combining multiple methods in a triangulation approach allows for greater accuracy in measuring and identifying a complex phenomenon such as camouflaging (Thurmond 2001). Participant report is needed to identify intention to camouflage, behavioural observation to identify how successful that camouflaging is, and measures of cognitive traits and autistic characteristics to identify how much the person is camouflaging their underlying ‘autistic-ness’ and how they do or do not achieve this. Methods for measuring behavioural camouflaging, and cognitive and autistic-like traits, already exist or have been proposed (Dean et al. 2017; Lai et al. 2017; Livingstone and Happé 2017); however until now, no self-report measures of camouflaging behaviours have been developed.

## Camouflaging Across the Dimensions

Autism is a dimensional characteristic; traits are distributed across the entire population, but with a cut-off point at the extreme end requiring clinical identification and support (Constantino 2011; Ruzich et al. 2015; Skuse et al. 2005). All individuals in the general population have some level of autistic traits, and those with an above average number may also camouflage these to varying extent. Camouflaging is similar to impression management, where behaviours which occur in front of others are manipulated in order to make a better impression (Leary and Kowalski 1990). Autistic individuals engage in impression management to a lesser degree than non-autistic individuals (Cage et al. 2013). The combination of underlying autistic characteristics and extent of (successful) camouflaging produces an external ‘autistic’ presentation, with corresponding variation in general functioning (Livingston and Happé 2017). Thus, it is important to develop measures of camouflaging that are appropriate for both autistic and non-autistic populations.

## The Present Study

A psychometrically sound self-report measure of camouflaging behaviours is needed to improve current understanding of the nature, causes and consequences of social camouflaging. Furthermore, existing methods of measuring camouflaging behaviours have not been validated in both autistic and non-autistic populations.

The aim of this study is therefore to develop, psychometrically evaluate, and validate a self-report measure of social camouflaging behaviours (henceforth referred to as the Camouflaging Autistic Traits Questionnaire; CAT-Q), appropriate for both autistic and non-autistic populations.

### Development

Preliminary items for the CAT-Q were developed from qualitative responses to a previous study, and were added to and refined by all the authors and several external experts.

### Psychometric Evaluation

Exploratory and confirmatory factor analyses were used to identify, refine, and test the underlying factor structure of the CAT-Q in two separate samples. Multi-group measurement invariance analyses were used to compare the underlying factor structure in the male and female autistic and non-autistic samples.

Internal consistency of the measure was estimated using Cronbach’s alpha, and test–retest reliability was established by re-sending the CAT-Q to a subsample of 30 autistic participants approximately 3 months after they first completed the survey.

Convergent validity of the new measure was determined by comparing camouflaging scores with scores on theoretically related constructs (Cronbach and Meehl 1955). Individuals with more autistic-like traits are likely to camouflage those traits to a greater extent, although this has not been tested empirically before. Camouflaging has also been associated with increased social anxiety and general anxiety, and decreased wellbeing, in qualitative reports (Hull et al. 2017), as well as with increased depression in quantitative research (Cage et al. 2017; Lai et al. 2017). Accordingly, convergent validity was explored by testing the correlation between camouflaging and autistic-like traits, social anxiety, general anxiety, wellbeing, and depression.

## Methods

### Participants

Validation of the CAT-Q was conducted in autistic and non-autistic samples which were recruited separately. Autistic participants were recruited via social media, through the Cambridge Autism Research Database (CARD), and through word-of-mouth. Non-autistic participants were recruited via social media and through word-of-mouth. Participants who self-reported as autistic were asked to detail the type of diagnosis, (e.g. Autism, Asperger’s Syndrome, Autism Spectrum Disorder), the age they were diagnosed, and the type of healthcare professional who diagnosed them. Those who reported being self-diagnosed were automatically excluded from the study and did not complete any further questions. All participants were at or above the legal age to give informed consent on their own behalf in the UK (16 years).

Of those autistic participants who reported the age they were diagnosed, 12% were diagnosed in childhood (0–17 years) and 72% were diagnosed in adulthood (18 years and over). Of those diagnosed in childhood, 38% were diagnosed by a psychiatrist, 25% by a clinical psychologist, 8% by other specialists including neurologists and specialist nurses, 5% by a multi-disciplinary team, 2% by a Speech & Language Therapist, 2% by their school, and 2% by a paediatrician. Of those diagnosed in adulthood, 55% were diagnosed by a clinical psychologist, 35% by a psychiatrist, 3% by a multi-disciplinary team, 3% by other specialists, 0.7% by a Speech and Language Therapist, 0.7% by a GP, and 0.3% by an occupational therapist.

In the autistic sample, 14% were aged 16–25, 23% were aged 26–35, 20% were aged 36–45, 13% were aged 56–65, 3% were aged 66–75, and 0.3% were aged 75 or over. In the non-autistic sample, 59% were aged 16–25, 16% were aged 26–35, 8% were aged 36–45, 9% were aged 46–55, 6% were aged 56–65, 1% were aged 66–75, and 0.2% were aged 75 or over (proportions may not add up to 1 due to rounding).

### Measures

#### Camouflaging Autistic Traits Questionnaire (CAT-Q)

The measure’s operationalisation of social camouflaging is based on the analysis and theoretical model described in the qualitative study by Hull et al. (2017). Items for the CAT-Q were identified through multiple routes. A previous study which asked for autistic adults’ experiences of camouflaging (Hull et al. 2017) also asked participants to describe specific behaviours they used while camouflaging. These responses were refined to produce a list of behaviours reflecting the two core components of camouflaging identified previously: compensation (i.e. finding ways around the social and communication difficulties associated with autism), and masking (i.e. hiding aspects of one’s autistic presentation, or presenting a non-autistic persona to others). Additional camouflaging behaviours were suggested by autism experts, including researchers, clinicians, and autistic adults who were consulted directly.

Once the behaviours were identified, items that described them, including reverse-coded items describing the opposite of these behaviours, were developed. Items were removed or added to ensure there was a roughly even number tapping into ‘compensation’ and ‘masking’. A total of 48 items were produced for inclusion in the study. Participants responded using a seven-point Likert scale, from ‘Strongly Disagree’ to ‘Strongly Agree’ with each statement.A total of 832 participants (354 adults with autism and 478 adults without autism) completed the CAT-Q.

#### Broad Autism Phenotype Questionnaire (BAPQ; Hurley et al. 2007)

A 36-item self-report measure of traits associated with the broader autism phenotype (BAP). BAP characteristics are associated with greater genetic liability for autism, and are found across the population and at especially high levels in relatives of those with an autism diagnosis. Scores for the total questionnaire and three sub-factors (Aloofness, Pragmatic Language, and Rigidity) are averaged across the 36 items in the total questionnaire and 12 in each factor, to produce values in a range of 0–6. A total of 744 participants (299 autistic and 445 non-autistic) completed the BAPQ. The BAPQ has good sensitivity (Sasson et al. 2013) and specificity (Hurley et al. 2007). Internal consistency of the BAPQ in the current study (total sample) was high (α = 0.96). Although the BAPQ was initially developed as a measure of autistic-like traits in relatives of those with autism, it has also been used to measure autistic-like traits in autistic and non-autistic groups (Ingersoll et al. 2011; Nishiyama et al. 2014; although see Piven and Sasson 2014 for an evaluation of this approach). In this case we included the BAPQ as a measure of autism-related characteristics, rather than as a screening tool for autism. Mean BAPQ scores were compared for autistic and non-autistic samples and were found to be significantly different (t[743] = 21.23, p < .001, d = 1.56), with means of 4.31 (SD = 0.69) for autistic participants and 3.18 (SD = 0.73) for non-autistic participants. This suggests that, although the BAPQ was designed for relatives of those with autism, there were no ceiling effects in the autistic sample.

#### Social Anxiety Scale (LSAS; Liebowitz 1987)

A 24-item self-report questionnaire measuring social anxiety in the general population. The scale requires participants to imagine being in different social situations (such as talking to a sales assistant in a shop) and asks how much fear they would experience and how much they would avoid the situation. The LSAS has demonstrated good test–retest reliability and discriminant validity (Baker et al. 2002). A total of 708 participants (284 adults with autism and 424 adults without autism) completed the LSAS. In the total sample of this study, internal consistency was high (α = 0.97).

#### Warwick–Edinburgh Mental Wellbeing Scale (WEMWBS; Tennant et al. 2007)

A 14-item self-report questionnaire measuring general wellbeing in the last 2 weeks. The WEMWBS has demonstrated acceptable validity and reliability (Trousselard et al. 2016). A total of 713 participants (289 adults with autism and 424 adults without autism) completed the WEMWBS. Internal consistency in the total sample was high (α = 0.92).

#### Patient Health Questionnaire (PHQ-9; Kroenke et al. 2001)

A 9-item self-report questionnaire of depressive symptoms in the last 2 weeks, with a clinical cut-off point of 10 for moderate depression. The PHQ-9 has demonstrated good sensitivity and specificity for depressive symptoms (Kroenke et al. 2001). The PHQ-9 was only administered to autistic individuals. A total of 290 autistic participants completed the PHQ-9. Internal consistency in the autistic sample was acceptable (α = 0.89).

#### Generalised Anxiety Disorder (GAD-7; Spitzer et al. 2006)

A 7-item self-report measure of generalised anxiety symptoms in the last 2 weeks. The GAD-7 has a clinical cut-off point of 10 points and demonstrates good sensitivity and specificity (Spitzer et al. 2006). The GAD-7 was only administered to autistic individuals. A total of 289 autistic participants completed the GAD-7. Internal consistency in the autistic sample was high (α = 0.92).

### Procedure

Participants followed a link to the online survey, hosted by Qualtrics, where they read the information sheet and, after contacting the researchers to answer any questions, completed a consent form. They then completed demographic questions and questionnaires.

Participants who had given contact details to researchers were contacted again 3 months later to ask them to re-take the questionnaire for the purpose of estimating test–retest reliability. At that time, adverts were also placed on social media inviting autistic participants who had previously completed the survey to complete it again.

### Analyses

All analyses were performed in R (R Core Team 2013).

The total sample was split in two, with the first half utilised for exploratory factor analysis to identify an initial factor structure from which a 25-item final scale was produced (‘exploratory sample’; N = 402), and the remainder utilised for confirmatory factor analysis (‘confirmatory sample’; N = 430). These two samples had comparable levels of autistic-like traits; however the confirmatory sample was significantly younger on average (partial η^2^ = 0.13), and contained proportionally more males (Cramer’s V = 0.12), than the exploratory sample.

#### Exploratory Factor Analysis

Principle components analyses using oblique rotation were performed on the total exploratory sample (N = 402), and separately in the autistic (N = 200) and non-autistic (N = 202) subsamples. Retention of items was based on combined evaluation of the scree plot, following Cattell (1966); eigenvalues over 1.0; and parallel analysis techniques to model factor structure (Hayton et al. 2004). Items with loadings below 0.40, or with cross-loadings of greater than 0.40 were excluded.

#### Confirmatory Factor Analysis

Diagonally Weighted Least Square Means (WLSM) estimators were used to take into account the ordinal nature of the Likert-based responses (DiStefano and Morgan 2014; Wang and Cunningham 2005). The key indices used to assess goodness-of-fit were Comparative Fit Index (CFI), where values of 0.95 or greater indicate good fit; Root Mean Square Error of Approximation (RMSEA), where values of 0.06 or lower indicate acceptable fit; and Standardised Root Mean Square Residual (SRMR), where values of 0.08 or lower indicate acceptable fit (Hu and Bentler 1999).

#### Multi-group Measurement Invariance

The total sample was recombined and multi-group measurement invariance analysis used to determine whether the same latent variables were measured across four groups: male autistic, female autistic, male non-autistic, and female non-autistic. Participants who identified as a non-binary gender or did not report their gender were excluded from this analysis (n = 92).

Tests of measurement invariance involve the comparison of multiple, nested models (Sass 2011) measuring: (1) Configural Invariance (whether factor structure is equal across groups); (2) Metric Invariance (whether item loading on each factor is equal across groups); (3) Scalar Invariance (item intercepts are equal across groups); and (4) Residual Invariance (item residuals are equal across groups). Each model is compared to the previous in a forward approach to first establish invariance across groups, and then test whether non-invariance has been identified at each additional level. ∆CFI of less than 0.01 is generally used as the most reliable marker of invariance, as Χ^2^ values can be influenced by sample size (Cheung and Rensvold 2002). Diagonally Weighted Least Square Means (WLSM) estimators were again used, and robust statistics are reported for all results. Satorra-Bentler scaled corrections for multiple comparisons were used.

#### Reliability and Validity

Internal consistency, test–retest reliability, and convergent validity of the final scale were assessed in a subset of the total sample who had also provided complete responses to at least one of the other measures in the study (N = 706; Autistic N = 306, Non-Autistic N = 400). Internal consistency was measured using Cronbach’s alpha, and test–retest reliability using Pearson’s r and intra-class coefficients (ICCs). Two-way consistency ICC was used to evaluate absolute consistency between the first and second completion of the questionnaire, (McGraw and Wong 1996), with unity reflecting complete consistency on all items between time one and time two. Values of 0.50 to 0.75 indicate moderate reliability, while values of 0.75 and above indicate good reliability (Koo and Li 2016).

Convergent validity was assessed using correlations between total CAT-Q and factor scores, and measures of autistic-like traits, social anxiety, well-being, generalised anxiety, and depression.

## Results

The characteristics of the total sample and all subsamples are described in Table 1.

**Table 1:** Sample characteristics

	Total sample	Autistic subsample	Non-autistic subsample	Exploratory subsample	Confirmatory subsample
N (male/female/other gender/not stated)	832 (300/434/46/52)	354 (108/179/17/50)	478 (192/255/29/2)	402 (139/246/17/0)	430 (161/188/29/52)
Mean age in years (SD)	36.01 (14.84)	41.93 (13.55)	30.24 (13.72)	37.02 (15.02)	35.15 (14.21)
Age range in years	16–82	18–75	16–82	16–82	16–72
Mean age at autism diagnosis (range)	–	34.2 (2–66)	–	34.47^a^ (2–66)	33.82^a^ (3–66)
Native language = English	617	244	373	346	271
Employed full- or part-time	308	135	173	182	126
Student	257	36	221	123	134
Retired or homemaker	62	43	19	39	23
Unemployed or unable to work	86	64	22	42	44

NB: some participants chose not to answer some demographic questions

^a^Value for autistic participants only

### Exploratory Analyses

Parallel analysis suggested four factors, but examination of the scree plot and eigenvalues suggested that three common factors best fit the data across the autistic, non-autistic, and combined samples, in addition to being a simpler structure. The three factors were labelled Compensation (strategies used to actively compensate for difficulties in social situations), Masking (strategies used to hide autistic characteristics or portray a non-autistic persona), and Assimilation (strategies that reflect trying fit in with others in social situations). These three factors accounted for 38% of variance in the autistic subsample, 41% of variance in the non-autistic subsample, and 45% of variance in the combined exploratory sample. Factor correlations were medium-to-high (Cohen 1988) between all factors in all samples (Table 2).

**Table 2:** Factor correlations in autistic, non-autistic and combined (Com) samples

	Compensation			Masking			Assimilation		
	Autistic	Non-Autistic	Com	Autistic	Non-autistic	Com	Autistic	Non-autistic	Com
Compensation	–	–	–	0.5	0.47	0.39	0.44	0.58	0.66
Masking	0.5	0.47	0.39	–	–	–	0.21	0.39	0.33
Assimilation	0.44	0.58	0.66	0.21	0.39	0.33	–	–	–

Items that loaded onto one of the three factors at or above the critical value of 0.40 in both the autistic and non-autistic subsamples, and in the combined sample, were identified. These were reduced to twenty-five items based on the highest factor loadings, which resulted in a total of 8 items each in the Masking and Assimilation factors, and 9 items in the Compensation factor. Table 3 presents the mean scores and internal consistencies of the factors and total scale across the autistic, non-autistic, and combined samples. Autistic participants scored significantly higher than non-autistic participants on the Total CAT-Q (*t* [401] = 12.98, *p* < .001; partial η^2^ = 0.30) and Compensation (*t* [401] = 11.90, *p* < .001; partial η^2^ = 0.26), Masking (*t* [401] = 2.19, *p* = .03; partial η^2^ = 0.01), and Assimilation factors (*t* [401] = 16.35, *p* < .001; partial η^2^ = 0.40). Factor loadings on all three factors in the final, 25-item Camouflaging Autistic Traits Questionnaire (CAT-Q) are detailed in Table 4.

**Table 3:** CAT-Q total and factor scores in the autistic (N = 200) and non-autistic (N = 202) subsamples and the combined exploratory sample (Com; N = 402)

Scale	No. of items	Mean (SD)			Internal consistency (Cronbach’s α)		
		Autistic	Non-autistic	Com	Autistic	Non-autistic	Com
Total	25	4.79 (0.99)	3.48 (1.04)	4.13 (1.21)	0.91	0.93	0.94
Compensation	9	4.42 (1.31)	2.89 (1.27)	3.65 (1.50)	0.88	0.90	0.92
Masking	8	4.55 (1.35)	4.29 (1.10)	4.42 (1.24)	0.87	0.84	0.86
Assimilation	8	5.29 (1.15)	3.32 (1.27)	4.30 (1.56)	0.86	0.89	0.93

Raw Scores have been rescaled to reflect the 7-Point Likert Scale

**Table 4:** Factors loadings of the 25-Item CAT-Q in autistic, non-autistic and combined (Com) exploratory subsamples

Item	Factors								
	Compensation			Masking			Assimilation		
	Autistic	Non-autistic	Com	Autistic	Non-autistic	Com	Autistic	Non-autistic	Com
When I am interacting with someone, I deliberately copy their body language or facial expressions	**0.48**	**0.60**	**0.58**	0.17	0.08	0.15	0.00	0.06	0.01
I learn how people use their bodies and faces to interact by watching television or films, or by reading fiction	**0.73**	**0.76**	**0.77**	0.09	0.04	0.06	– 0.10	0.01	0.02
I have tried to improve my understanding of social skills by watching other people	**0.73**	**0.73**	**0.73**	0.04	0.03	0.04	– 0.13	0.02	0.04
I will repeat phrases that I have heard others say in the exact same way that I first heard them	**0.59**	**0.53**	**0.57**	– 0.28	0.15	– 0.06	0.22	0.09	0.18
I practice my facial expressions and body language to make sure they look natural	**0.51**	**0.61**	**0.61**	**0.32**	0.15	0.25	– 0.03	0.04	– 0.03
I have spent time learning social skills from television shows and films, and try to use these in my interactions	**0.83**	**0.72**	**0.79**	– 0.01	– 0.01	0.01	– 0.03	0.15	0.09
In my own social interactions, I use behaviours that I have learned from watching other people interacting	**0.76**	**0.74**	**0.73**	0.02	0.13	0.10	– 0.03	0.01	0.04
I have researched the rules of social interactions (for example, by studying psychology or reading books on human behaviour) to improve my own social skills	**0.61**	**0.41**	**0.56**	0.06	0.02	0.03	– 0.01	0.15	0.14
I have developed a script to follow in social situations (for example, a list of questions or topics of conversation)	**0.53**	**0.46**	**0.48**	0.02	0.17	0.09	0.28	0.16	0.28
I monitor my body language or facial expressions so that I appear relaxed	0.03	**0.32**	0.17	**0.85**	**0.60**	**0.75**	0.02	0.04	– 0.02
I adjust my body language or facial expressions so that I appear relaxed	0.08	**0.31**	0.22	**0.79**	**0.56**	**0.69**	– 0.06	0.06	– 0.04
I monitor my body language or facial expressions so that I appear interested by the person I am interacting with	0.11	0.22	0.16	**0.71**	**0.52**	**0.66**	0.12	0.09	0.03
I adjust my body language or facial expressions so that I appear interested by the person I am interacting with	0.06	0.23	0.15	**0.74**	**0.57**	**0.69**	0.07	0.08	0.02
I don’t feel the need to make eye contact with other people if I don’t want to (Reversed)	– 0.02	– 0.23	– 0.18	**0.59**	**0.30**	**0.52**	– 0.01	0.14	0.01
In social interactions, I do not pay attention to what my face or body are doing (Reversed)	0.00	0.07	0.03	**0.82**	**0.47**	**0.69**	– 0.11	0.12	– 0.01
I always think about the impression I make on other people	– 0.01	0.02	– 0.08	**0.39**	**0.61**	**0.52**	0.17	0.05	0.13
I am always aware of the impression I make on other people	– 0.10	– 0.06	– 0.16	**0.44**	**0.63**	**0.54**	0.01	– 0.16	– 0.08
I rarely feel the need to put on an act in order to get through a social situation (Reversed)	0.00	– 0.13	– 0.08	0.24	0.19	0.21	**0.56**	**0.69**	**0.71**
When talking to other people, I feel like the conversation flows naturally (Reversed)	– 0.08	– 0.11	– 0.03	– 0.14	– 0.02	– 0.13	**0.70**	**0.75**	**0.85**
When in social situations, I try to find ways to avoid interacting with others	0.01	0.28	0.14	– 0.21	– 0.21	– 0.18	**0.66**	**0.66**	**0.75**
In social situations, I feel like I’m “performing” rather than being myself	0.04	0.04	0.06	0.12	0.27	0.11	**0.70**	**0.57**	**0.75**
I have to force myself to interact with people when I am in social situations	0.06	0.17	0.10	– 0.05	– 0.11	– 0.05	**0.72**	**0.72**	**0.77**
In social situations, I feel like I am pretending to be “normal”	0.00	0.21	0.09	0.19	0.18	0.13	**0.65**	**0.58**	**0.74**
I need the support of other people in order to socialise	0.08	**0.31**	0.16	– 0.11	– 0.04	– 0.07	**0.60**	**0.52**	**0.66**
I feel free to be myself when I am with other people (Reversed)	– 0.07	– 0.15	– 0.10	0.19	0.10	0.09	**0.63**	**0.69**	**0.81**

Loadings of 0.30 and greater are in bold

### Confirmatory Analyses

Confirmatory factor analysis was performed on the confirmatory sample (N = 419; Autistic N = 150, Non-Autistic N = 269); the results for the autistic, non-autistic, and combined group analyses for the total scale are reported in Table 5.

**Table 5:** Fit of 25-Item Full CAT-Q Scale across autistic (N = 154), non-autistic (N = 276) and Combined confirmatory samples (Com; N = 430)

Sample	Χ^2^	Df	CFI	RMSEA (90% CI)	SRMR
Autistic	596.947*	272	0.970	0.056 (0.050–0.063)	0.075
Non-Autistic	619.099*	272	0.983	0.046 (0.041–0.051)	0.058
Com	969.527*	272	0.980	0.052 (0.048–0.055)	0.057

Robust Statistics are reported

*CFI* Comparative Fit Index, *RMSEA* root mean square error of approximation, *90% CI* 90% confidence intervals, *SRMR* standardised root mean square residual

*p < .0001. Χ^2^ = Chi squared; Df = degrees of freedom

**Fig. 1:**
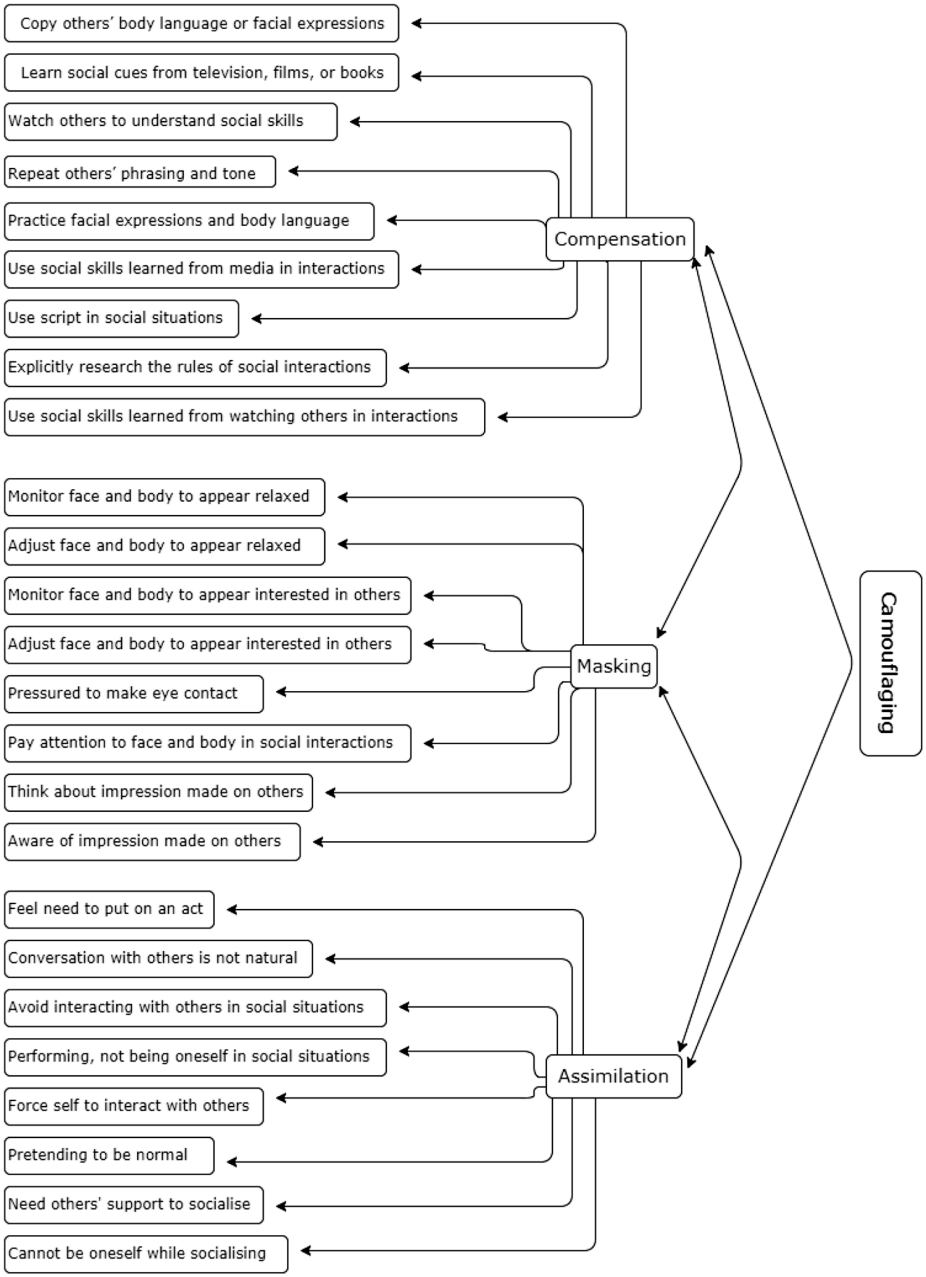
Social Camouflaging model

Overall the model fit was acceptable; CFI values were above 0.95, and RMSEA and SRMR values were well within the recommended range in all three samples. The model tested is detailed in Fig. 1.

### Invariance Analyses

Measurement invariance (as demonstrated by ∆CFI < 0.01) was found for item loadings (Model 2), intercepts (Model 3), and residuals (Model 4) between the autistic and non-autistic male and female samples (as shown in Table 6). Model fit was close to or within acceptable limits for all models. It was concluded that the CAT-Q demonstrates strict invariance between autistic and non-autistic males and females.

**Table 6:** Multi-group measurement invariance model comparison (autistic male N = 107; autistic female N = 181; non-autistic male N = 192; non-autistic female N = 256)

Model	Χ^2^	∆Χ^2^	Df	CFI	∆CFI
1. Configural invariance	2434.22	–	1088	0.947	–
2. Metric invariance	2353.93	80.29	1154	0.953	0.006
3. Scalar invariance	2628.31	272.38	1220	0.945	0.008
4. Residual invariance	2856.15	227.84	1295	0.939	0.006

Robust statistics are reported

*Χ*^2^ chi squared, ∆*Χ*^2^ chi square difference, *Df* degrees of freedom, *CFI* Comparative Fit Index, ∆*CFI* CFI difference

### Reliability and Validity

Reliability and validity of the finalised, 25-item scale were assessed in a subset of the total sample that had also provided complete responses to at least one of the other measures included (N = 706; Autistic N = 306, Non-Autistic N = 400).

### Reliability

High internal consistency was found for the total scale (Cronbach’s α = 0.94), and the Compensation (α = 0.91), Masking (α = 0.85), and Assimilation (α = 0.92) factors. Correlations between each factor and the total score were calculated using item-total correlation, and the corrected factor-total correlations in the total sample were: Compensation α = 0.705; Masking α = 0.483, Assimilation α = 0.627.

Test–retest reliability was calculated in a subsample of 30 autistic participants, who completed the CAT-Q again online three months after initial testing. This subsample was significantly older on average than the total autistic sample (F[1, 331] = 12.61, p < .001; mean difference = 9.23 years [SE = 2.6]). However, there was no significant difference in the distribution of genders (Male, Female, Other Gender, and not stated) (Χ^2^ [4] = 1.66, p = .80), and no significant difference in mean Total BAPQ score (t[299] = 0.55, p = .59) between this subsample and the total autistic sample. Good stability was found, as measured by Pearson’s r and intra-class correlations (ICC) for the total scale and the Compensation factor, while moderate stability was found for the Masking and Assimilation factors (Table 7). No significant difference between scores at Time 1 and Time 2 was found (F[1, 29] = 0.23, p = .63).

**Table 7:** Test–retest reliability of Camouflaging Autistic Traits Questionnaire and factors in autistic subsample (N = 30)

	Pearson’s r	ICC[C,1]	95% CI
Total CAT-Q	0.77	0.77	0.73, 0.79
Compensation factor	0.78	0.77	0.72, 0.82
Masking factor	0.70	0.70	0.63, 0.76
Assimilation factor	0.73	0.73	0.67, 0.78

### Validation

Correlations were performed between the total and factor CAT-Q scores, and scores on autistic-like traits (total BAPQ score and subscale scores), social anxiety (total LSAS score), wellbeing (total WEMWBS score), generalised anxiety (total GAD-7 score), and depression (total PHQ-9 score) in order to investigate convergent validity. Results in the autistic and non-autistic samples are detailed in Table 8. Generalised anxiety and depression scores were available for autistic participants only.

**Table 8:** Correlations between CAT-Q Total and factor scores and autistic traits (BAPQ), social anxiety (LSAS), wellbeing (WEMWBS), depression (PHQ), and generalised anxiety (GAD) for the autistic (N = 306) and non-autistic (N = 400) subsamples

	Total BAPQ	BAPQ: Aloof	BAPQ: pragmatic language	BAPQ: rigidity	Total LSAS	WEMWBS	PHQ	GAD
Autistic								
CAT-Q total	0.34***	0.24***	0.33***	0.28***	0.44***	− 0.16*	0.28***	0.35***
Compensation	0.21***	0.08	0.27***	0.18**	0.30***	− 0.02	0.18**	0.25***
Masking	− 0.03	− 0.07	− 0.03	0.01	0.19**	− 0.02	0.16**	0.20***
Assimilation	0.72***	0.63***	0.62***	0.54***	0.60***	− 0.37***	0.35***	0.41***
Non-autistic								
CAT-Q total	0.67***	0.58***	0.56***	0.54***	0.60***	− 0.43***	–	–
Compensation	0.54***	0.42***	0.52***	0.44***	0.46***	− 0.31***	–	–
Masking	0.32***	0.24***	0.24***	0.32***	0.35***	− 0.24***	–	–
Assimilation	0.78***	0.77***	0.62***	0.59***	0.69***	− 0.53***	–	–

*p < .05, **p < .01, ***p < .001

The total CAT-Q score and all CAT-Q factors were significantly positively correlated with autistic-like traits and social anxiety in autistic and non-autistic samples, with the exception of the Masking factor, which was not significantly related to autistic-like traits in the autistic sample. The total CAT-Q and all CAT-Q factors were significantly negatively correlated with wellbeing in the non-autistic sample; however, in the autistic sample, only total CAT-Q and the Assimilation factor were significantly negatively correlated with wellbeing. Depression and generalised anxiety were only measured in the autistic sample; both of these were significantly positively correlated with total CAT-Q and all its factors.

## Discussion

This study psychometrically tested the newly developed Camouflaging Autistic Traits Questionnaire (CAT-Q) in autistic and non-autistic samples. Exploratory factor analysis identified a three-factor structure, consisting of Compensation (strategies used to compensate for social and communication difficulties), Masking (strategies used to present a non-autistic or less autistic persona to others), and Assimilation (strategies used to fit in to uncomfortable social situations). The structure of the refined, 25-item CAT-Q (see Online Appendix 1) was corroborated through confirmatory factor analysis, and measurement invariance was established between all four groups, suggesting that the CAT-Q is appropriate for use in clinical and non-clinical populations, and that scores can be compared between males and females. The CAT-Q demonstrated acceptable to good internal consistency and reliability over a period of 3 months. However, as the test–retest reliability analyses were conducted only in the older autistic sample, we report these findings as preliminary and suggest future research replicates these analyses in more diverse autistic and non-autistic samples.

The factors of Compensation and Masking reflect the two components of camouflaging proposed in a previous conceptual model derived from qualitative research (Hull et al. 2017). The third factor (‘Assimilation’) represents attempts to blend in to social situations in which the individual is uncomfortable, without letting others see this discomfort. These motivations for camouflaging have been described in previous research, although not extensively (Hull et al. 2017; Tint and Weiss 2017). The strategies within the Assimilation factor included avoiding social situations or managing them with the help of others, alongside items reflecting the feeling of not being one’s self during interactions. The factor reflects comments made by autistic adults that they often choose to camouflage in situations where they do not know others well, whereas they feel free to be themselves while alone or with trusted others (Hull et al. 2017).

The model tested here provided a good fit in both autistic and non-autistic samples. Total CAT-Q score was positively correlated with autistic-like traits in both samples, suggesting that the higher level of autistic-like traits a person has, the more they will camouflage those traits, regardless of autism diagnosis. As high-level, successful camouflaging may result in missed clinical diagnoses (Tierney et al. 2016), the CAT-Q could be used to identify camouflaging behaviours in individuals considered at-risk for autism, but who do not currently meet diagnostic criteria. Measurement invariance analyses also demonstrated that the underlying structure of the CAT-Q is comparable in male and female autistic and non-autistic samples; in other words, the CAT-Q measures the same latent constructs in both genders and diagnostic groups. However, autistic participants scored significantly higher than non-autistic participants on the total CAT-Q and all three factors in the exploratory sample, demonstrating that the CAT-Q measures behaviours that are more common in individuals who have been diagnosed with autism spectrum conditions.

The Masking factor demonstrated the smallest difference between autistic and non-autistic samples in this analysis, suggesting that there may be more overlap between these two groups than for the other factors. Masking may be less specific to autism than the other components of camouflaging, and may reflect more general self-presentation or impression-management strategies applied to autistic characteristics. However, further research is needed to directly compare masking strategies and other self-presentation strategies in autistic and non-autistic samples to determine similarities and differences. In the autistic sample, masking was not significantly correlated with autistic-like traits, suggesting that it may be a response to the identification of being autistic rather than to the presence of specific autistic characteristics; in contrast, a significant positive relationship between the two was observed for the non-autistic sample, suggesting that the two groups may have been using masking strategies in response to different motivations.

Previous research suggested that camouflaging in autistic adults may be associated with poor mental health outcomes, especially anxiety, depression, and generally poor quality of life (Cage et al. 2017; Hull et al. 2017; Lai et al. 2017). The positive correlations between the CAT-Q and measures of social anxiety, anxiety, and depression, and the negative correlation between the CAT-Q and wellbeing, support this idea and offer convergent validation of the measure. Greater total camouflaging appears to be associated with poorer mental health outcomes overall, although interestingly the Compensation and Masking factors were not significantly associated with wellbeing in the autistic sample. This may reflect individual differences in the impact or success of camouflaging; previous research found that associations between camouflaging and negative outcomes were stronger for autistic men than women (Lai et al. 2017). Further assessment of gender differences and other individual differences in camouflaging behaviours and their association with wellbeing and mental health in this sample is currently underway.

### Strengths and Limitations

A significant strength of this approach is that the items were developed based on information from autistic people themselves, describing their own experiences of camouflaging. This ensures that behaviours which may not have been previously identified as part of social camouflaging by non-autistic clinicians and researchers can be measured. The CAT-Q can be used in combination with observed behavioural and cognitive measures of camouflaging to assess all aspects of this complex phenomenon. It may also have clinical implications to identify levels of camouflaging along with other clinical information, including those derived from current autism diagnostic measures, to enhance the sensitivity and specificity of clinical diagnosis, formulation, and support planning; however, the clinical utility requires further clinical research to establish.

In addition, the CAT-Q does not require an official diagnosis of an autism spectrum condition for camouflaging behaviours to be assessed, as the underlying structure shows invariance between autistic and non-autistic populations. This addresses some issues in current autism research, especially that criteria for autistic participants may be based on an overly restricted and potentially inaccurate operational definition of autism. Even if autism diagnostic criteria change in the future, use of the CAT-Q should not vary between clinical and non-clinical groups. The CAT-Q has demonstrated measurement invariance between male and female participants, enabling comparison across genders in future research.

This study is not without its limitations. First, although the BAPQ has demonstrated validity and reliability in clinical and non-clinical samples (Ingersoll et al. 2011; Nishiyama et al. 2014), it was developed for use with relatives of those with an autism diagnosis. Therefore we are cautious about using BAPQ scores as a measure of autistic traits in clinical and general population samples (Piven and Sasson 2014). In future, to accurately examine how camouflaging is related to autistic traits, the CAT-Q should be compared to a measure of autistic traits which has been explicitly developed for use in autistic populations, for example, the severity score of the ADOS-2.

Second, no behavioural measure of social ability was included in the study. Individuals with greater social skills are less likely to need to camouflage in the first place, and may do so more effectively than those with poorer social skills. Further research is needed to identify the extent to which social skills predict camouflaging behaviours, which will have implications regarding prevailing social skills training in autistic individuals. There was also no objective validation of self-reported autism diagnosis. However, only participants who reported receiving a diagnosis from a healthcare professional were included in the autistic sample. Third, responses on the PHQ-9 and GAD-7 were not available for non-autistic participants as these data were collected as part of a separate project; the relationship between camouflaging and depression and anxiety should therefore also be examined in non-autistic adults.

Fourthly, the self-report CAT-Q only measures individuals’ own reflections/perceptions of their camouflaging behaviours, and is thus limited in its use to those who are able to reflect on their own behaviours and provide insight to their motivations. The CAT-Q may therefore not be useful for autistic individuals with language difficulties or intellectual disability. By combining this measure with behavioural or informant-report measures of camouflaging, estimates of camouflaging behaviours in those who have less insight or ability to communicate it can also be obtained.

Fifthly, the CAT-Q was created mainly based on reflections from autistic adults, and was psychometrically examined and validated in the present adult sample, in which a substantial proportion of the autistic participants received their diagnoses in adulthood instead of childhood. Hence, although the validity and potential clinical utility are likely ensured in autistic adults, in particular those who are diagnosed in adulthood (Lai and Baron-Cohen 2015), it is still unclear whether the psychometric properties and potential utilities hold for adolescents and older children, with or without autism, or for those with intellectual disability. Further testing of the CAT-Q in samples of varying ages and abilities, including adults who were diagnosed in childhood, should be conducted to measure its factor structure, validity and reliability across these groups. As the confirmatory sample contained more males than females, these analyses should also be replicated in a gender-matched sample. Finally, although the validation of the CAT-Q supports previous research suggesting camouflaging is associated with poorer wellbeing and mental health outcomes, only correlational relationships were identified. Longitudinal or intervention researches are necessary to confirm the causal nature of these relationships, and to establish the mechanisms and individual characteristics that may predict outcomes of camouflaging.

## Conclusions

The CAT-Q is a valid and reliable self-report measure of adults’ social camouflaging behaviours, suitable for use in autistic and non-autistic male and female populations. It can be used in research settings to quantify camouflaging behaviours and compare between groups; in clinical settings as a potential screening tool for individuals who may be missed under current autism diagnostic criteria because they camouflage; and by autistic and non-autistic people to aid identification of beneficial or harmful behaviours they use in social situations. Further validation of the CAT-Q in more diverse samples is encouraged in the future, alongside comparison with existing measures of camouflaging and broader social skills.

## Electronic supplementary material

Below is the link to the electronic supplementary material.

Online Appendix 1 (DOCX 14 KB)
