# Supplementary material for: Development and Validation of the Camouflaging Autistic Traits Questionnaire (CAT-Q)
Source: J Autism Dev Disord. 2018 Oct 25;49(3):819–33. doi: 10.1007/s10803-018-3792-6 (PMC6394586; doi:10.1007/s10803-018-3792-6)
Supplement: Supplementary file 1 — Online Appendix 1 (DOCX 14 KB) [file 10803_2018_3792_MOESM1_ESM.docx]

Appendix 1

**Camouflaging Autistic Traits Questionnaire (CAT-Q)**

Please read each statement below and choose the answer that best fits your experiences during social interactions.

| Strongly Disagree (1) | Disagree (2) | Somewhat Disagree (3) | Neither Agree nor Disagree (4) | Somewhat Agree  (5) | Agree  (6) | Strongly Agree  (7) |
| --- | --- | --- | --- | --- | --- | --- |

1. When I am interacting with someone, I deliberately copy their body language or facial expressions
2. I monitor my body language or facial expressions so that I appear relaxed
3. I rarely feel the need to put on an act in order to get through a social situation*
4. I have developed a script to follow in social situations (for example, a list of questions or topics of conversation)
5. I will repeat phrases that I have heard others say in the exact same way that I first heard them
6. I adjust my body language or facial expressions so that I appear interested by the person I am interacting with
7. In social situations, I feel like I’m ‘performing’ rather than being myself
8. In my own social interactions, I use behaviours that I have learned from watching other people interacting
9. I always think about the impression I make on other people
10. I need the support of other people in order to socialise
11. I practice my facial expressions and body language to make sure they look natural
12. I don’t feel the need to make eye contact with other people if I don’t want to*
13. I have to force myself to interact with people when I am in social situations
14. I have tried to improve my understanding of social skills by watching other people
15. I monitor my body language or facial expressions so that I appear interested by the person I am interacting with
16. When in social situations, I try to find ways to avoid interacting with others
17. I have researched the rules of social interactions (for example, by studying psychology or reading books on human behaviour) to improve my own social skills
18. I am always aware of the impression I make on other people
19. I feel free to be myself when I am with other people*
20. I learn how people use their bodies and faces to interact by watching television or films, or by reading fiction
21. I adjust my body language or facial expressions so that I appear relaxed
22. When talking to other people, I feel like the conversation flows naturally*
23. I have spent time learning social skills from television shows and films, and try to use these in my interactions
24. In social interactions, I do not pay attention to what my face or body are doing*
25. In social situations, I feel like I am pretending to be ‘normal’

**Scoring:**

All items are scored 1-7, with higher scores reflecting greater camouflaging. Items with an asterisk (*) should be reverse scored.

**Factors:**

Compensation = 1, 4, 5, 8, 11, 14, 17, 20, 23

Masking = 2, 6, 9, 12, 15, 18, 21, 24

Assimilation = 3, 7, 10, 13, 16, 19, 22, 25
